# Imaging-Assisted Large-Format Breast Pathology: Program Rationale and Development in a Nonprofit Health System in the United States

**DOI:** 10.1155/2012/171792

**Published:** 2012-12-17

**Authors:** F. Lee Tucker

**Affiliations:** Virginia Biomedical Laboratories, LLC, P.O. Box 510, Wirtz, VA 24184, USA

## Abstract

Modern breast imaging, including magnetic resonance imaging, provides an increasingly clear depiction of breast cancer extent, often with suboptimal pathologic confirmation. Pathologic findings guide management decisions, and small increments in reported tumor characteristics may rationalize significant changes in therapy and staging. Pathologic techniques to grossly examine resected breast tissue have changed little during this era of improved breast imaging and still rely primarily on the techniques of gross inspection and specimen palpation. Only limited imaging information is typically conveyed to pathologists, typically in the form of wire-localization images from breast-conserving procedures. Conventional techniques of specimen dissection and section submission destroy the three-dimensional integrity of the breast anatomy and tumor distribution. These traditional methods of breast specimen examination impose unnecessary limitations on correlation with imaging studies, measurement of cancer extent, multifocality, and margin distance. Improvements in pathologic diagnosis, reporting, and correlation of breast cancer characteristics can be achieved by integrating breast imagers into the specimen examination process and the use of large-format sections which preserve local anatomy. This paper describes the successful creation of a large-format pathology program to routinely serve all patients in a busy interdisciplinary breast center associated with a community-based nonprofit health system in the United States.

## 1. Introduction

Significant among the advances of the last 3 decades in breast cancer diagnosis and management are the broad access to mammographic service screening by asymptomatic individuals [1–5], diagnosis by minimally invasive needle biopsy techniques, and the widespread acceptance of breast conservation surgery. Surgical management, specifically the complete surgical removal of all detectable breast carcinoma, remains the preferred first therapeutic step for the majority of men and women with Stage 0–III breast cancer [6, 7]. Breast-conserving procedures, as an evidence-based alternative to mastectomy, have gained widespread acceptance since the NSABP B-04 and B-06 trials over 30 years ago [8, 9]. Pathologic examination of resected tissue continues to serve as the definitive means of establishing adequacy of breast conservation surgery (BCS) with the reporting of surgical margin status. As such, margin evaluation remains a key data point in the clinical decision of whether to administer radiation therapy postoperatively [10] or to perform an additional excision to obtain negative margins. Neither the NSABP B-04 or B-06 trials defined margin negativity beyond the simple absence of neoplasm on the margin itself, so although margin negativity was required for accrual into the BCS-radiation therapy arm, proximity of tumor or tumor subtype to surgical margins was neither reported nor controlled. 

The last 30 years have yielded confusing and contradictory information regarding the risk of local or systemic recurrence and survival following BCS. Numerous authors have sought to identify variables useful in the prediction of breast cancer recurrence or at least to serve as criteria for surgical reexcision. Many of these variables have been drawn from the characteristics of the neoplasm itself or the proximity of the neoplasm to surgical margins. Investigators have reported the relationship between recurrence (or the presence of residual cancer in reexcision specimens) and the proximity of invasive cancer to negative BCS margins [11–13], the number of involved margins [14], multifocality, and the presence of an extensive intraductal component (EIC) associated with an invasive cancer [15, 16]. Unfortunately, many reports attempting to correlate recurrence risk with proximity to surgical margins failed to define margin status relative to duct carcinoma *in situ* (DCIS) [17–19], and so it was not known to what extent margin positivity for DCIS may have contributed to observed recurrence rates. Gradually, DCIS was identified as an independent risk factor in breast cancer recurrence after BCS. More recent studies have demonstrated varying degrees of correlation between the overall extent of DCIS [20–22], nuclear grade [23], and proximity to surgical margins [24–27] and risk of breast cancer recurrence. In some series, over 50% of women undergoing breast conservation surgery required additional surgery to achieve satisfactory margin status [28]. Most of these reports have been inspired by one or more persistent clinical questions, relevant to the clinical management and prognosis of a patient diagnosed with breast cancer (Table 1).

It has been difficult to draw a clear consensus from much of the breast cancer recurrence literature published over the last 3 decades; however some general conclusions are reasonable. Positive margins for both DCIS and invasive cancer place a patient at high risk for local recurrence and are to be avoided. Secondly, negative margins are not a guarantee against recurrence, yet increasing margin width crudely correlates with decreasing risk of recurrence by reducing the likelihood of residual carcinoma in the adjacent breast. Greater extent of DCIS [29] and higher nuclear grade are positively correlated with recurrence, even with negative margins (21, 23). Despite these broad generalizations, which in retrospect appear intuitive, a high degree of concordance across these studies is not observed with covariables such as margin width of DCIS and risk of local failure [30] or DCIS extent and risk of recurrence. The lack of consensus in this literature is frustrating to surgeons and oncologists alike who seek reproducible data to support evidence-based management protocols. It seems obvious that margin evaluation is not an exact science, but how much of the conflicting data is related to the biology of breast cancer itself? Some authors have pointed to difficulties in standardizing, optimizing, and reporting breast specimen examinations by pathologists [31, 32]. This general lack of concordance suggests that the pathology data collection itself is not optimized for reproducibility; a reasonable conclusion considering the conventional methods of gross specimen examination used to report breast cancers is essentially unchanged from the preimaging era [33]. 

The literature addressing tumor attributes and risk of recurrence mostly relies upon data abstracted from pathology reports based on traditional methods of gross and microscopic examination. In the vast majority of these laboratories, surgical specimens are sectioned to conform to industry-standard 25 mm × 175 mm glass microscope slides. Most reports do not describe centralized or expert remeasurement of reported breast cancer metrics. Measurement methodologies for DCIS extent vary from laboratory to laboratory and are often not specified in the publication. Lester et al. [34] describe a protocol sanctioned by the College of American Pathologists for the examination of specimens from patients with DCIS. The protocol is a welcome effort to assist pathologists in the identification and reporting of DCIS in resected tissue. The authors appropriately enumerate the varied methods used by pathologists to measure DCIS extent but no recommendation was made to standardize the process or correlate imaging data into specimen examination. Commonly, pathologists measure the largest single focus on an individual slide or the span of DCIS across a single slide and report the result as DCIS extent. Other pathologists calculate extent by counting the number of slides involved with DCIS. Some pathologists add the gross section width (e.g., 3 mm, 5 mm) of involved slides to estimate cumulative DCIS extent [35], a practice found by Dadmanesh et al. to underestimate extent in 72% of cases [36]. Rarely, a pathologist will attempt to correlate histopathology with imaging studies and measure DCIS extent across images using the locations of slides positive for DCIS. Reconciliation of presurgical imaging characteristics with pathologic measurements of size, extent, or margin status prior to finalizing the pathology report seems to be the exception, rather than the rule. 

In clinical practice, margin evaluation is now routinely facilitated by surgical specimen orientation. Reporting of 6-axis BCS margins is standard practice in most centers; however numerous technical limitations plague the process. Breast tissue is inherently pliable, and the dimensions of the specimen and associated neoplasia can change dramatically with changes in patient position from mammogram to MRI, operating suite and pathology work station. Pathologists or their assistants typically apply multicolored inks to the specimen circumference to approximate the orienting clips or sutures placed surgically; however this process typically occurs without surgeon validation of the designated margins. The resulting margin boundaries (e.g., anterior-medial or posterior-lateral) may or may not represent the surgeon's view of the same boundaries. The risk for the patient is that a reexcision of a close or involved margin may not align with the margin as identified by the pathologist essentially leaving a critical margin unresected. 

Despite remarkable advances in breast imaging technology since the early screening trials including digital mammography, 3D automated ultrasound, digital tomosynthesis, magnetic resonance imaging (MRI), and breast-specific gamma imaging (BSGI), the techniques employed by pathologists to grossly examine resected breast tissue have changed little, save for the measurement of margins on oriented specimens. Specimen radiographs obtained for wire-localization BCS procedures are usually available for pathologist review; however the contribution of these images to the gross and microscopic examination is only occasionally documented in pathology reports. The degree to which wire-localization radiographs guide the pathologist's determination of cancer size, extent, and proximity to margins is unknown. In a study of 135 BCS procedures for DCIS, mammography was found to significantly underestimate the extent of DCIS as measured pathologically even when performing complete specimen radiography and extensive tissue sampling [37]. Compared to mammography, presurgical MRI has significantly higher sensitivity in the detection of DCIS [38]; however presurgical MRI studies are typically not available to pathologists at the time of specimen gross examination. Lacking knowledge of the imaging extent and distribution of a neoplasm in three dimensions (as is possible with MRI), gross surgical specimens are sectioned without reference to the axis of greatest cancer extent or mammographically occult encroachment on surgical margins. Pathologists intentionally select 6-axis sections for microscopic study when a grossly palpable or visible area of concern is identified, when wire-localization images suggest margin compromise or when a surgeon identifies margins of concern. Otherwise, sections are randomly selected based on gross inspection to represent 6-axis margins. The specimen is further morcellated until the individual tissue blocks conform to the industry-standard tissue cassette, an area no larger than 25 mm × 35 mm.

Improved characterization of DCIS extent and margin status has resulted from protocols requiring submission of 100% of lumpectomy tissue, notably the serial subgross techniques utilized by Holland et al. [39], MacDonald et al. [21, 23], Cheng et al. [20], Kato et al. [40], and Sigal-Zafrani et al. [41]. Three-dimensional reconstruction techniques to improve margin evaluation were reported by Mai et al. [42] and Ichihara et al. [43]. Techniques referred to as serial sequential sectioning employed by Grin et al. [35] and Dadmanesh et al. [36] approach the goal of optimizing cancer extent measurements. These techniques typically maintain the spatial orientation of the tissue sections for subsequent three-dimensional reconstruction. Unfortunately, these methods require that additional breast tissue is trimmed from the submitted tissue blocks so they conform to standard tissue processing methods. The result is a diminution or loss of local breast and tumor anatomic relationships. In some centers, mammographic examinations are routinely performed on intact tissue slices [22, 23, 35]; in others, radiographic examination of gross specimen sections is either performed on a subset of sections or not at all. 

A significant limitation to the accurate reporting of DCIS extent and margin status results from poor information flow among the breast care team members at the time of surgery. Modern imaging techniques provide the breast imager and surgeon with an increasingly lucid presurgical depiction of the extent and distribution of breast neoplasia [43]. Functional imaging, particularly breast MRI, gives insight into the anatomic distribution of neoplasm in three dimensions [44, 45] and can identify disease, particularly DCIS extent beyond the sensitivity and specificity of mammography or ultrasound [46, 47]. Many studies have attempted to evaluate the sensitivity or specificity of mammography, ultrasound, and MRI using reported pathologic tumor characteristics as the reference standard. Most of the published imaging-pathology correlation studies import tumor measurements directly from cancer registry or pathology reports. The data thus obtained is subject to all of the limitations associated with conventional pathologic techniques. As an example, Ichihara et al. [43] found MRI was not only more sensitive than mammography in detection of DCIS (88% versus 27%) but maximum extent of DCIS measured by mammogram (90 mm) or MRI-detection (110 mm) far exceeded maximum pathology extent measurements (25 mm). Schouten van der Velden et al. [45] in comparing reported pathology cancer size measurements of 49 cancers with estimates by mammogram and MRI found agreement within 5 mm in only 27% of mammogram and 38% of MRI studies. Some authors regard the discrepant size and extent measurements as evidence that MRI overestimates the extent of breast carcinoma [44] and raise concerns of unnecessary or additional surgery [47, 48].

Notably absent from the current debate on the sensitivity and specificity of breast imaging technology and the clinical utility of breast MRI as a presurgical planning procedure is a discussion of the limitations of the pathologic techniques used to validate modern imaging studies. The practical limitations of conventional breast pathology reporting techniques [31, 49, 50] are summarized in Table 2.

Prior to the widespread clinical use of breast MRI, breast pathologists using large-format contiguous histologic sections measuring up to 8 × 10 cm visualized breast cancer in three dimensions [33, 51] and improved correlation between imaging and pathologic measurements of cancer size and extent [49, 52]. Subsequent reports have confirmed the clinical advantages of large-format breast specimen processing with improved imaging-pathology correlation of cancer distribution and optimized evaluation of surgical margins [50, 53, 54]. The suitability of these techniques to the community hospital setting and the benefit to both imager and pathologist were described by Biesemier and Alexander [55] and Méchine-Neuville et al. [56].

## 2. Materials and Methods

The image-guided large-format breast pathology (LBP) initiative at Carilion Clinic necessitated preimplementation development of several key program elements (Table 3). 

### 2.1. Planning-Phase Knowledge Base and Budget Development

The specimen processing methods at Carilion Clinic were developed over a six-month period. They were adapted from procedures in place at Falun Central Hospital, Falun, Sweden and Central Virginia Pathology Consultants, Lynchburg, Virginia following site visits to those locations. Capital and operating budgets were prepared, and all laboratory capital and operating expenses were funded exclusively from laboratory operating revenue. A separate cost center was not created within the histology laboratory for the large-format program; however expenses attributed to histology labor and consumables were recorded for analysis of incremental costs in comparison with conventional pathology processing. Specimen radiography and radiograph interpretation were performed in the breast imaging department in a cost center created for the purpose of supporting the large-format pathology program. The technical and professional component billing and revenue capture for specimen radiography and interpretation using CPT codes 76098-TC and 76098-26 were booked to the specimen radiography cost center. 

### 2.2. Specialized LBP/MRI Interdisciplinary Conference

This weekly conference, attended by breast imagers, surgeons, and pathologists, was created to optimize surgical outcomes through the continuous correlation of large-format pathology with clinical findings and all pre- and postimaging data, including MRI. As an adjunct to the existing weekly interdisciplinary breast pretreatment planning conference, this conference was designed to accomplish two primary goals. A detailed postsurgical analysis of tumor size, extent, multifocality, and proximity to surgical margins was performed by correlating all presurgical breast imaging studies with subgross and histopathologic findings. This was accomplished with real-time projection of large-format pathology slides alongside MRI and other projected presurgical imaging studies. Presurgical imaging estimates of cancer extent, geographic distribution, and proximity to anatomic landmarks were reviewed and revised with large-format pathologic validation. Final surgical margin measurements were scrutinized in view of presurgical procedure planning goals and intraoperative surgical findings. Secondly, presurgical planning was conducted with a goal of optimizing the utilization and outcomes of breast-conserving procedures whenever possible. The conference differed from the usual presurgical planning conference by leveraging the team's experience gained from the post-surgical large-format-imaging correlations. Breast MRI was routinely performed on most cases diagnosed and treated in the LBP era and was generally not available in this breast program prior to implementation of LBP. 

### 2.3. Surgical Pathology Gross Specimen Protocol

All mastectomy, lumpectomy, and reexcision specimens are included in the large-format program with specialized processing procedures designed for each specimen type. Mastectomy specimens were phased-in beginning August 2004 in order to gain experience with the technique. Breast-conserving specimens and reexcisions were added to the program in November 2004. Nearly 400 surgical specimens representing between 250 and 275 new breast cancer diagnoses are accessioned and processed annually.

Upon receipt in the surgical pathology suite, all specimens are checked for proper surgical orientation, and any deficiencies identified are promptly corrected by the surgeon. All surgical margins are marked before sectioning with India ink with a discrete color coded to each of six axial margins. Mastectomy specimens are sectioned in the sagittal plane at uniform 5 mm increments with preservation of medial-to-lateral sequence and cephalocaudad orientation. Initially, 100% of each mastectomy specimen was submitted for specimen radiography. Experience permitted introduction of time and cost conserving measures so that approximately one-half to two-thirds of each specimen was submitted for radiography, focusing on the location of previous biopsy or lumpectomy sites and any additional guidance provided by breast surgeons and imagers. Breast-conserving specimens and reexcisions are inked for margins and sectioned at 5 mm increments. The plane of sectioning varies with the requirements of the case. Occasionally, demonstration of maximum extent of disease or multifocality in one plane is of paramount importance. In these cases, the plane of sectioning is guided by image interpretation of size, extent, and multifocality, and sections are typically made in the coronal (frontal) plane. In cases where the status of chest wall or anterior cutaneous margins (or both) are of particular concern or the sagittal plane shows maximum extent, the sagittal plane is selected for sectioning and large-format section submission. This approach allows convenient correlation of sagittal large histology sections with MRI sagittal-reconstructed images. All BCS and reexcision specimens are entirely submitted for specimen radiography.

**Figure 1:**
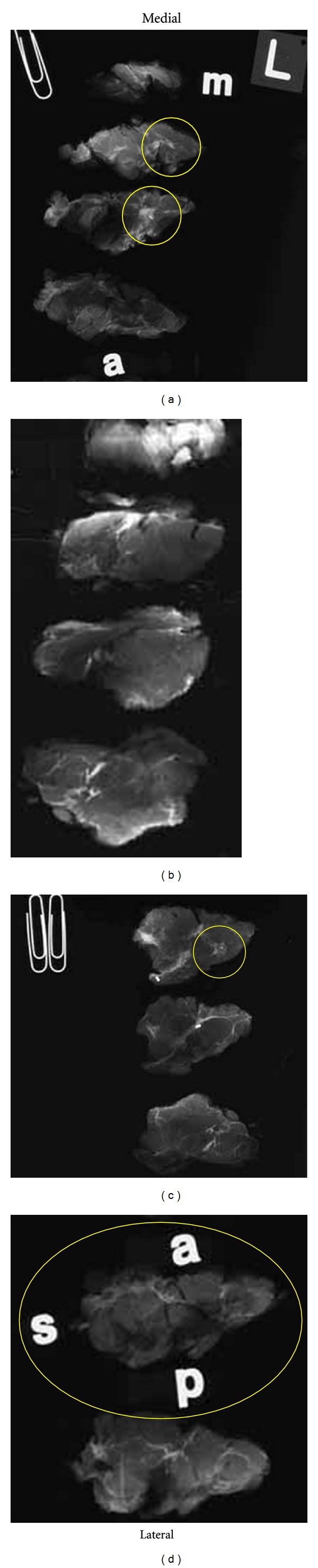
Specimen radiographs of a lumpectomy from a left breast sectioned at 5 mm intervals in the sagittal plane. Sections are sequentially oriented from medial (a) to lateral (d). Yellow circles correspond to marks made by a breast imager following review and correlation of relevant imaging studies to denote significant radiographic findings such as calcification, mass density, architectural distortion, ultrasound findings and enhancement on MRI. The imager selectively marks sections to demonstrate maximum size and extent of neoplasia and close proximity to margins with the expectation that the pathologist will provide histopathologic correlation of identified imaging findings.

Orientation of all specimens is maintained with the use of radioopaque alphabet characters to denote axis margins in each plane as well as the sequence of sections, for example, “superficial to deep” in the coronal plane or “medial to lateral” in the sagittal plane. Tissue slices are placed on previously exposed radiographic film with orienting characters and transported expeditiously to the breast imaging facility for radiography. Following specimen radiography and radiographic interpretation (vide infra), the specimen with accompanying radiographs is returned for pathologic examination with a “Large-Format Specimen Checklist” detailing the clinical and specimen radiographic findings in written narrative and using template diagrams of both breasts. This worksheet communicates the clinical and radiographic findings of concern with specific questions to be addressed pathologically. Imaging abnormalities visible mammographically are marked directly on the films with a wax pencil as a guide for histopathologic section submission (Figure 1). The location and extent of ultrasound or MRI-detected findings, not visible mammographically or with specimen radiography, are marked on the specimen radiographs. Section(s) best demonstrating critical findings such as proximity to margins or maximum extent of neoplasm are encircled for possible large-format submission. 

**Figure 2:**
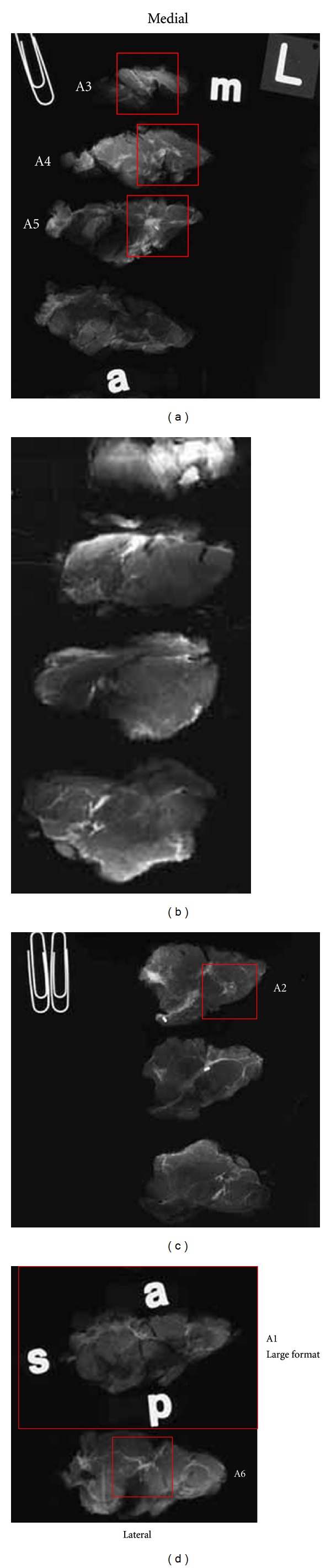
Specimen radiographs from lumpectomy shown in Figure 1 marked after pathologic examination. Red rectangles denote tissue blocks removed for microscopic examination. Block A1 consists of a large-format tissue section with circumferential surgical margins in the sagittal plane. Orientation is maintained on the radiograph with opaque letters “a” (anterior), “s” (superior), and “p” (posterior). The inferior margin is opposite superior (unmarked). Blocks A2–A6 are conventionally sized. Medial and lateral surgical margins are represented by conventional sections A3 and A6, respectively. Blocks A2 and A4 were removed to address specific findings identified by the breast imager.

Following traditional pathologic specimen examination with gross inspection and palpation, sections are selected for histologic study to incorporate gross pathologic findings as well as findings of concern communicated by imagers and surgeons (Figure 2). Microscopic evaluation of surgical margins is typically accomplished with a combination of both large-format and conventionally sized tissue blocks. Large sections usually permit margin evaluation along 100% of the circumference of a BCS specimen and are selected to encompass critical margins whenever close proximity to a surgical line of excision is suspected. Margins perpendicular to the large-format plane of section are usually submitted as conventional tissue blocks. Orientation of all conventionally sized margin sections is perpendicular to the specimen surface to allow measurement of margin width to the nearest whole millimeter. The precise location of these perpendicular margins is selected with guidance from both imaging and clinical information and conventional pathologic examination. Reexcision specimens, including surgical shave excisions of biopsy cavities, are sectioned and oriented perpendicular to the surgical margin so that margin distance from the cavity to the new surgical margins remains measureable. The anatomic location of all tissue blocks removed from the specimen is recorded in the gross dictation narrative of the pathology report as well as on the specimen radiographs which are preserved as a permanent record of pathology slide origin. This record is provided to the pathologist at microscopic sign-out as an aid in the reconstruction of the specimen in three dimensions.

### 2.4. Breast Imaging Department Specimen Radiography Protocol

Upon receipt in the imaging center, specimens are placed in an analog specimen radiography unit (Faxitron Bioptics, LLC). Specimen film images are examined by a breast imager, and all relevant mammograms, core biopsy specimen radiographs, wire-localization images, ultrasound, and MRI studies are reviewed. Mammographically detected lesions of concern are marked on the radiograph films, and the location of mammogram occult abnormalities identified with ultrasonography and MRI is encircled. To facilitate communication of clinical, presurgical imaging and specimen radiography findings to the pathologist, the “Large-format Specimen Checklist” is completed with reference to notations made by the imager on the specimen radiographs. A graphical depiction of the location and extent of clinical and imaging-suspected neoplasia is marked on a template bilateral breast diagram. A written narrative conveys clinical concerns and imager-initiated questions for pathologist reconciliation. This form is returned with the specimen and annotated specimen radiographs to the surgical pathology suite. 

### 2.5. Histopathology Laboratory Large-Format Specimen Procedures

**Figure 3:**
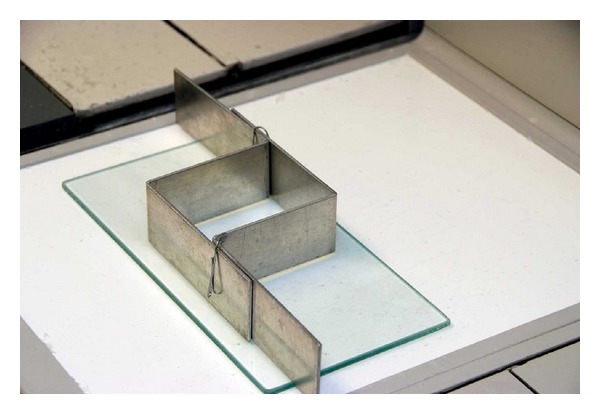
Adjustable embedding forms.

Upon receipt in the histopathology laboratory, conventional tissue blocks are separated from the large-format tissue blocks and separately processed in the routine manner. Large-format blocks are fixed for an additional 24–32 hours in 10% neutral buffered formalin. After fixation, all breast tissue is processed on automated processors (Tissue-Tek VIP, Sakura Finetek USA, Inc.). Paraffin embedding of large tissue sections requires reusable cassette forms fabricated on-site from 14 ga × 1 − 1/4′′ wide aluminum bar stock (Figure 3). Paraffin block dimensions are thereby conformable to the size of the tissue block and measure up to 8 × 10 cm. Sections are prepared at 3 to 4 microns on a Leica sliding microtome model SM2500 (Leica Microsystems). Slide staining is performed on an automated histology slide stainer with slide baskets customized to hold large-format glass slides (Shandon Varistain 24-4, Thermo Electron Corporation). 

### 2.6. Pathologist Signout and Reporting Procedures

Pathologists are provided with transcribed gross dictation, all slides including large-format slides, annotated specimen radiographs, and the “Large-format Specimen Checklist” completed by the breast imager. With attention to the specific questions communicated by the breast imager, histopathology slides and radiographs are analyzed to complete all relevant data fields following the College of American Pathologists' recommended synoptic reporting protocol. When maximum size of invasive carcinoma or extent of *in situ* carcinoma is represented by the large-format slide, that dimension is directly measured from the slide and reported. When clinical or imaging information indicates a greater size/extent than represented on the large-format slide, a three-dimensional reconstruction of tumor size/extent is performed. Following guidance provided by the breast imager on the large-format specimen worksheet, specimen radiographs are correlated with histopathology slides keyed to individual specimen radiographic images. Size and extent are either directly measured or calculated using the uniform 5 mm section thickness as a guide and reported to the nearest whole millimeter.

Margin evaluation of BCS specimens is accomplished with similar reference to imager guidance. In sagittal-plane sections, circumferential microscopic evaluation of margins is correlated between large-format slides and specimen radiographs depicting anterior, inferior, posterior, and superior margins. Medial and lateral margin sections, whether conventional or of the large-format type, are similarly correlated with images. Proximity to each of six axial margins is measured to the nearest whole millimeter and recorded as 0 mm (positive), 1–10 mm in 1 mm increments, or >10 mm. Margin involvement of a boundary between two margins is reported as involvement of both margins at their junction, for example, “anterior-lateral” or “superior-medial.” A series of 135 consecutive lumpectomy specimens with a diagnosis of DCIS without invasive carcinoma were analyzed for margin status using the above techniques. Ninety-two specimens processed following the LBP protocol including presurgical MRI were compared to forty-three specimens processed using conventional pathologic techniques without presurgical MRI. The surgical reexcision rate and breast conservation rate in each category were retrieved from cancer registry data, and the volume of each lumpectomy specimen was computed with dimensions obtained from the gross pathology specimen description.

### 2.7. Technical Expense Analysis

The average number of conventional pathology blocks produced per mastectomy and lumpectomy specimen was tabulated for a total of 100 resections. The block totals were compared between cases submitted in calendar year 2004 with conventional pathology processing and in 2005 with large-format processing. An average of one large-format block per case was submitted in 2005. 

Technical expense for labor and materials associated with the processing of breast pathology specimens were calculated for 50 consecutive cases in calendar year 2005. Histotechnologist time devoted to the processing of conventional and large-format breast tissue from the time of receipt to delivery of stained and coverslipped slides was separately recorded for conventional and large-format processed tissue. Total technical time, expressed in fractional hours in each category was multiplied by the average hourly wage, including all benefits, of the histotechnologists directly involved with the project to arrive at total labor expense (TLE) for conventional and large-format processing. Unit labor expense per slide was calculated by dividing TLE by the total number of finished slides produced in each category.

The direct cost of consumables per slide followed a similar methodology. All materials used in processing and slide production were allocated to each category as appropriate. For liquid reagents and stains shared in processing, a *pro-rata *allocation between categories was estimated using the surface area of conventional versus large-format slides multiplied by the number of slides in each category. Total cost of consumables in each category was divided by the number of slides produced to calculate consumable cost per completed slide for each group. Finally, TLE and material cost per finished slide in each category were combined to calculate total technical expense for both conventional and large-format slides. Amortization of capital equipment and indirect costs, such as overhead and staff training, were not included in the calculation of technical expense. Professional time devoted to the examination and reporting of conventional and large-format sections was not compared.

### 2.8. Comprehensive Breast Cancer Database Development

A searchable breast database was developed at the inception of the program using Microsoft Access (Microsoft Corporation). All data collections and analyses were performed in compliance with Institutional Review Board approved research protocols. Separate pages in the database were created for clinical, mammography and ultrasound, MRI, and pathology data fields. The database was jointly maintained by cancer registry and Information Technology Department staff. Training of cancer registry and breast center staff in the population of data fields and search methodology was conducted by Information Technology staff.

## 3. Results

### 3.1. Surgical Margin Evaluation

**Figure 4:**
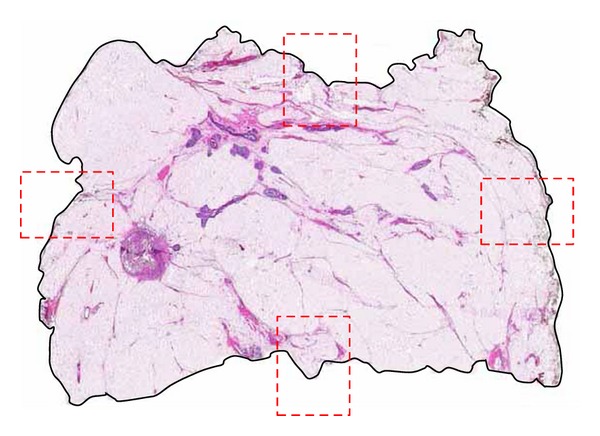
Large-format histologic section of a lumpectomy specimen. The circumferential large-format surgical margin is outlined with solid black line. Conventional margin evaluation would consist of four axis margin sections in one plane represented here by broken red rectangles (H&E).

**Figure 5:**
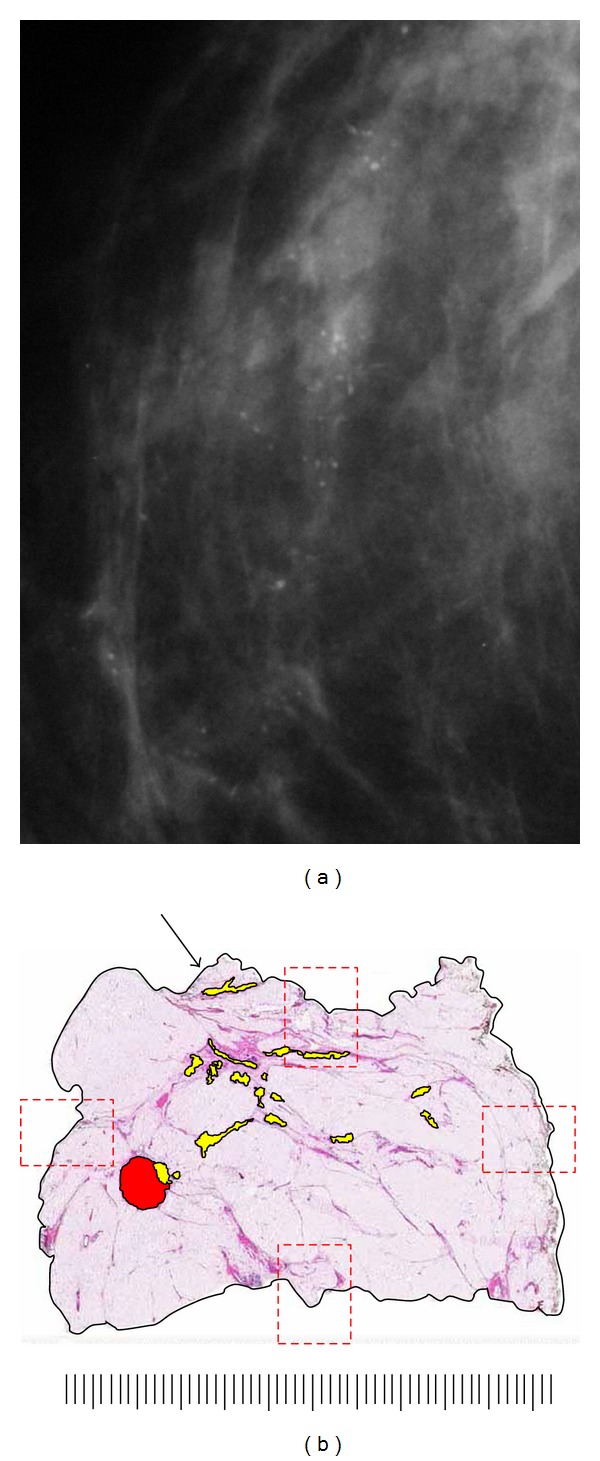
Analysis of case shown in Figure 4. (a) Asymptomatic 53-year-old woman with linear-casting calcifications on screening mammogram presented with no palpable abnormality. (b) Stereoguided biopsy showed grade 2 DCIS with necrosis. Core biopsy site is highlighted in red. DCIS is highlighted in yellow with maximum extent of 46 mm. Specimen on gross examination was minimally fibrotic and negative for a palpable lesion or gross evidence of DCIS. Specimen radiography guided selection of tissue blocks and large-format section to best demonstrate extent of neoplasm and proximity to superior margin (Arrow). Presurgical MRI was not performed. Conventional margin section selection guided only by gross inspection and palpation could have missed DCIS encroachment on superior margin which measured 1.5 mm. Scale: small divisions = 1.0 mm (H&E).

Conventional histopathologic evaluation of surgical margins is typically accomplished in BCS specimens with six tissue blocks, one selected in each margin axis. In any one plane of section, four conventionally sized tissue blocks are usually removed for microscopic analysis (Figures 4 and 5).

 The relationship between histologic section orientation and percent of the surface margin available for microscopic study in a hypothetical 8.0 cm diameter BCS specimen is presented in Table 4. If each of the conventional margin sections spans 1.0 cm of arc, approximately 16% of the circumference in one plane of section is available for microscopic study. Alternatively, if the margin sections each span 2.0 cm of margin circumference, the proportion represented rises to 32%. In contrast, the large-format method permits microscopic examination of 100% of the circumference of the selected plane of section of a BCS specimen. The margin evaluation of a three-dimensional resection specimen is more complex than the evaluation of margins in a single plane, however. En-face margin sampling theoretically permits the examination of a greater percentage of the specimen surface; however this method does not allow for the measurement of margin width. Perpendicular orientation of margin blocks facilitates measurement of margin distance at the expense of proportionate sampling of the specimen surface. As seen in Table 4, routine histologic sections of 4-micron thickness represent a very small percentage of the specimen surface area, even when compared with the amount of tissue theoretically available in a 3 mm thick paraffin block. The representative nature of margin evaluation, as evidenced by the small proportion of surface margin actually examined, illustrates the necessity of obtaining the greatest yield and relevance possible from each margin section removed from a specimen. In the LBP program, large-format section selection was guided by review of imaging and clinical data conveyed at the time of specimen gross examination. In approximately 10% of cases, two or more large-format sections were submitted to gain experience with the technique and to correlate the extent of neoplasia with MRI. With experience however, one optimally selected large-format section supplemented with conventionally sized blocks proved sufficient to evaluate margins, demonstrate maximum extent of neoplasia, and correlate with imaging studies. 

The influence of the LBP program on reported surgical margins and reexcision rate for DCIS was compared with preimplementation conventional pathologic reporting. The effect of the LBP initiative was evaluated in the context of a fully integrated interdisciplinary breast program characterized by meticulous presurgical planning, routine use of large-format sections, and correlative postsurgical interdisciplinary analysis of tumor size, extent, multifocality, and proximity to surgical margins. Adequacy of breast conserving surgery was expressed as margin width in millimeters exceeding a selected distance in each of six axes. The integrated LBP program with presurgical MRI is compared with conventional breast pathology without presurgical MRI in Table 5.

### 3.2. Ink Migration

**Figure 6:**
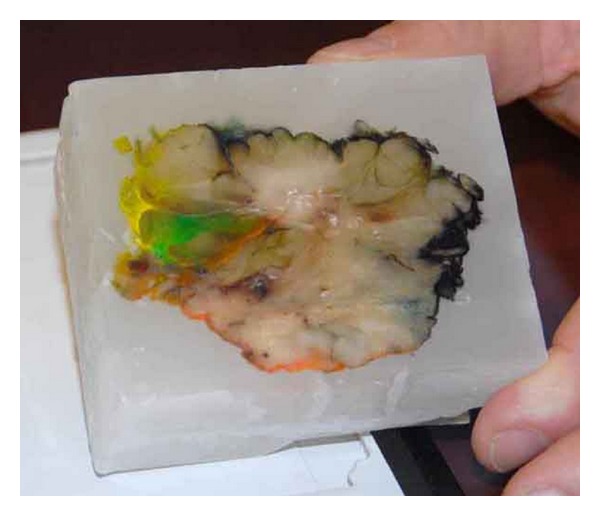
Paraffin block from lumpectomy specimen marked with India ink in multiple colors for orientation of surgical margins. Note migration of ink into interior of specimen.

**Figure 7:**
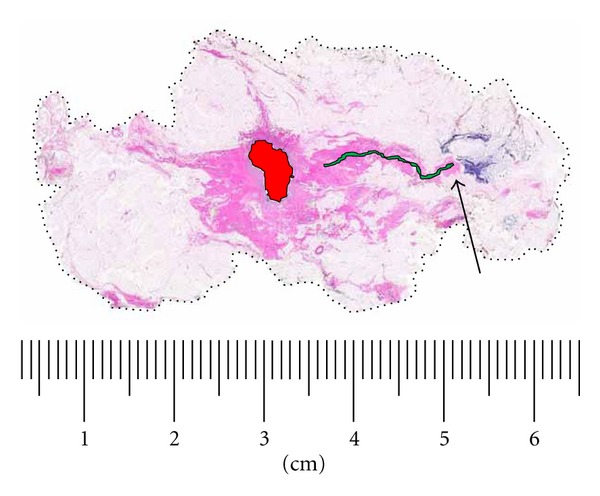
Large-format section of a lumpectomy specimen with circumferential surgical margin (broken line). Biopsy cavity is centrally located (solid red). Grade 2 DCIS (solid green) extends 15 mm from the cavity to within 9 mm of true surgical margin. Arrow marks migration of India ink into specimen to within 1 mm from DCIS. A conventional histologic section taken in this area could misrepresent the margin distance as 1 mm. This pitfall is easily avoided on the large-format slide. Scale: small divisions = 1.0 mm (H&E).

India ink applied to the surface of a breast specimen to mark the surgical margin may migrate into cracks and clefts in the specimen surface as noted by Campbell et al. [32] (Figure 6). On microscopic examination, the pathologist encountering an inked tissue edge on a conventional pathology slide may regard it as a true inked margin and measure margin width from a focus of cancer to the visible ink, unaware that the true margin is at greater distance (Figure 7). 

**Figure 8:**
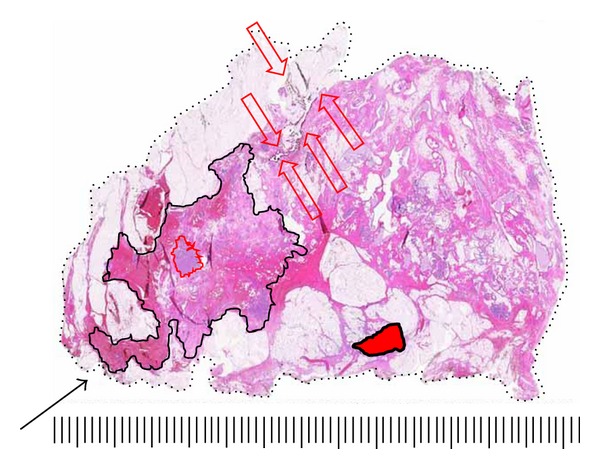
Large-format section of a lumpectomy specimen from a 72 year woman. India Ink migration into the interior of the specimen is marked with open red arrows. The true surgical margin is represented by broken black line. Grade 2 DCIS extending 35 m (solid black line) is present 2 mm from migrating ink. A small grade 1 invasive ductal carcinoma is outlined by a solid red line. A biopsy cavity is located lower-central (solid red fill). The biopsy was positive for DCIS. No residual DCIS was found within 12 mm of the biopsy site. The solid black arrow points to a positive surgical margin suspected on specimen radiograph which led to the submission of this section. Scale: small divisions = 1.0 mm (H&E).

The migration of India ink into the interior of breast surgical specimens is relatively common and appears to occur along fissures in the specimen exposed or created by the surgical procedure or specimen handling. With conventional histologic sections, tissue blocks removed from a surgical specimen exhibiting an inked tissue edge may be construed as a surgical margin. A potential for misinterpretation of the margin width exists if the tissue block was taken from an area of ink migration. In such a circumstance, the margin measurement would be lower than the true margin width. Preservation of the anatomy of the specimen with large-format sections permits clear identification of the peripheral margin contour so that ink migration into the interior of the tissue block is recognized and not confused with India ink on a true peripheral surgical margin (Figure 8).

### 3.3. Measurements of DCIS Extent

Imaging studies, particularly specimen radiographs, are correlated with histopathology in the determination of greatest extent of DCIS and maximum size of invasive carcinoma at the time of pathologist signout in all cases. In many but not all instances, large-format histologic sections provide the best depiction of maximum extent of disease. In such cases, extent can be measured directly from the large section slides themselves. Frequently, however, it is necessary to reconstruct the specimen in three dimensions. Three-dimensional reconstructions are made by correlating the annotated specimen radiographs with individual tissue blocks keyed to the radiographs. Reference is made to imaging estimates of tumor size and extent. In these reconstructions, calculations of DCIS extent are based on a combination of direct slide measurement and calculation of maximum distance of histologically confirmed DCIS across uniform 5 mm tissue slices. A comparison of DCIS extent measurements between conventional pathologic techniques and imaging-guided large-format breast pathology (LBP) is given in Table 6. 

**Figure 9:**
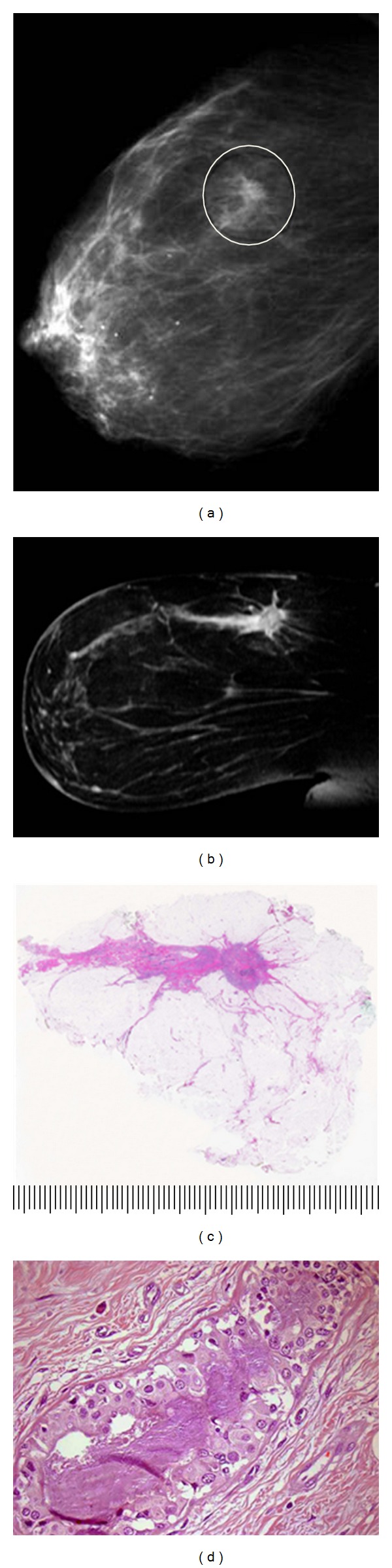
Seventy-one-year-old woman presenting with a palpable nodule of the right breast. (a) Right MLO mammogram with 14 mm stellate density. (b) MRI of right breast, sagittal reconstruction. The estimated overall extent of enhancing lesion 60 mm. (c) Large-format pathology slide of lumpectomy specimen, sectioned in the sagittal plane. Overall extent of DCIS is 60 mm with a superimposed 15 mm invasive ductal carcinoma corresponding to stellate density (H&E). (d) DCIS grade 3 located 2 mm from the anterior-superior surgical margin (H&E, 100x).

**Figure 10:**
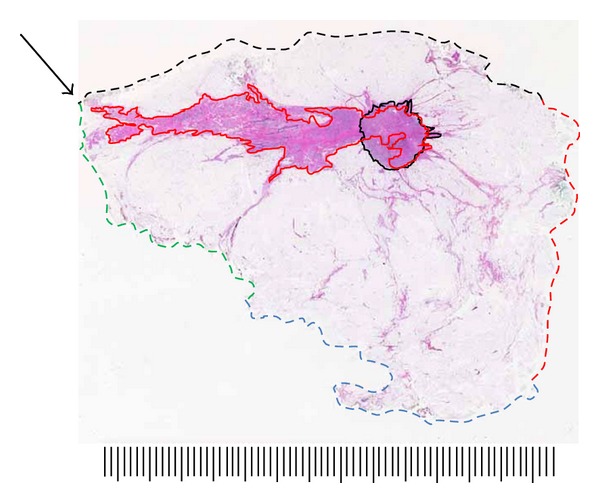
Large-format section of lumpectomy specimen from Figure 9. Analysis of neoplasm size, extent, and margin status. Invasive grade 2 ductal carcinoma is outlined with solid black line, 15 mm greatest dimension. Grade 3 DCIS extent outlined with solid red line, 60 mm overall. Circumferential margins marked with broken line. Specimen was sectioned in the sagittal plane to best evaluate critical anterior-superior margin. DCIS extends to within 2 mm of intersection of anterior and superior margins (arrow). Scale: small divisions = 1.0 mm (H&E).

A typical case depicting tumor size and extent measurement and margin analysis using the LBP technique is shown in Figures 9 and 10. In this example, the 71-year-old patient presented with a palpable 1-2 cm nodule of the upper-inner quadrant. Mammogram showed a BIRADS 4 stellate density 14 mm in diameter corresponding to the palpable lesion. Ultrasonography disclosed an 15 mm hypoechoic lesion amenable to ultrasound-guided core needle biopsy, which was positive for grade 3 invasive ductal carcinoma. Presurgical MRI obtained for surgical planning purposes confirmed the presence of the biopsied lesion and additionally demonstrated a 60 mm area of enhancement extending anteriorly and superiorly from the index lesion. The patient was offered and accepted breast conservation surgery. The specimen was sectioned in the sagittal plane to correlate with MRI sagittal-reconstructed images and to best demonstrate extent of MRI enhancement and proximity to anterior, posterior, superior, and inferior margins. Gross examination of the specimen disclosed a firm 15 mm nodule; however, most of the enhancing 60 mm MRI-detected lesion consisted of clinically, surgically, and pathologically impalpable grade 3 DCIS. Final margin analysis reported DCIS present 2.0 mm from the anterior-superior margin. All other margins were >10 mm for DCIS and invasive carcinoma.

### 3.4. Technical Expense Analysis

The adoption of LBP techniques incurs additional technical expense. The primary capital expense is for a sliding microtome which can be purchased new for $55,000–$65,000 (Leica Microsystems). Existing laboratories may configure their tissue processors and staining equipment to accommodate large-format specimens if excess capacity exists. Otherwise, conventional automated processors (e.g., Sakura Tissue-Tek VIP, $50,000) and stainers (Sakura Tissue-Tek DRS, $30,000) may be acquired for this purpose and are easily adapted to large sections.

The direct labor and materials expense for LBP is moderated by cost savings resulting from a reduction in the number of conventional pathology sections submitted per case (Table 7). The focused, image-guided nature of the LBP technique results in the elimination of low-yield “random” sections typically submitted in mastectomy and lumpectomy cases. The direct technical component labor and materials expense for conventional and large-format slides is given in Table 8. This expense calculation does not include indirect expenses such as allocated overhead and staff training or amortization of capital equipment. Also not included in the expense calculation is an allowance for additional professional time for LBP gross specimen examination by pathologists or pathology assistants. The net incremental technical expense per case is derived by subtracting the savings realized in reducing the number of conventional pathology sections per case from the increased expense associated with large section submission (Table 9).

## 4. Discussion

For nearly three decades, numerous reports have analyzed pathologist-derived data such as DCIS extent or distance to surgical margins to predict risk for cancer recurrence or provide an evidence-based rationale for surgical reexcision following breast conservation surgery. The pathologic methods used to generate this data have been poorly standardized and largely bereft of imaging correlation. The reproducibility of the methods typically used by pathologists to examine surgical specimens and report breast cancer attributes has been largely unchallenged until recently.

The analysis and reporting of surgical margins, as it is practiced today, remains a crude science. For example, DCIS may escape gross detection in the dense as well as fat-replaced breast. Without gross findings to provide guidance, margin sections are often randomly submitted without reference to imaging studies. On all but the very smallest of excisions, only a subset of the specimen surface is examined histologically. For these reasons, the typical margin analysis may not accurately and completely document proximity of neoplasm to the surface margin. To its credit, the 2005 International Consensus Committee Panel on Image-Detected Breast Cancer made general recommendations for the examination of surgical specimens including the routine use of correlative specimen radiography or ultrasonography [57]. The panel also recommended “rigorous and documented” specimen sampling to allow for a targeted return for additional sampling or, better still, processing of the specimen “in its entirety.” These recommendations, however welcome, have not resolved the barriers to information flow between imaging and pathology departments nor were they intended to address the additional costs associated with complete specimen processing. The panel did not reference large-format techniques as described by Tot and others [50, 56].

In clinical practice, less than optimal correlation occurs between imaging and pathologist-reported tumor characteristics. The College of American Pathologists, as part of its voluntary Q-Probes quality assessment program, recently reported a retrospective pathology-imaging correlation study from 48 institutions [58]. This study is an important step in the right direction but was restricted to self-reported pathology departmental processes from core needle biopsies and did not purport to evaluate routines involving surgical excision specimens. Even so, it provides valuable insight into the prevailing attitudes and practices among the subset of pathologists participating in the program. In the CAP Q-Probes study, most pathology departments (65%) did not have a formal mechanism in place to correlate imaging with core biopsy results. The frequency with which pathologists conduct formal imaging correlation with lumpectomy or mastectomy results prior to finalizing the pathology report is unknown and may be even lower. Many breast pathologists currently use specimen radiography to identify calcifications in surgical specimens, needle biopsy cores, or paraffin blocks; breast imager involvement with these investigations is variable and largely unknown. The imaging-guided large-format breast pathology techniques described here require imager involvement and eliminate barriers to the communication of relevant clinical and imaging data to the pathologist. These techniques involve the imager prospectively in the identification and reporting of multiple tumor foci, axis of greatest cancer extent, and the proximity of impalpable neoplasm to surgical margins. The addition of imaging guidance to the pathologist's examination is not only relevant but timely. The best opportunity for pathologist identification and anatomic localization of these findings comes before the specimen is morcellated and the 3D relationships are lost. Preservation of the breast anatomy in three dimensions is an essential aspect of this technique. Not only is the pathologist able to reconstruct the extent of the neoplasm in three dimensions, but the breast imager is better able to reconcile true cancer extent with imaging findings, especially MRI enhancing lesions. 

The long-recognized discordance between reported pathologic tumor characteristics and imaging estimates of tumor size and extent has persisted despite a better presurgical impression of the complexity of breast cancers in three dimensions. This discordance has been attributed by some to a lack of specificity on the part of the imaging studies themselves [44] without reference to the limitations inherent in the pathology techniques long held as the gold standard for measuring breast cancer characteristics. A formal validation study of breast MRI using benchmark large-format examination techniques is beyond the scope of this paper but could contribute significantly to our understanding of the sensitivity and specificity of presurgical MRI in predicting breast cancer extent and multifocality.

Although it is generally agreed that most breast cancer recurrences after BCS are related to residual cancer in the ipsilateral breast, large-format specimen analysis illustrates how complex the 3D architecture of breast cancer, particularly DCIS, can be. Clearly, any margin sampling method short of including 100% of a specimen surface will be representative by nature. To improve the sensitivity of margin analysis, a strategy to improve the yield and specificity of margin section selection is required. At the present time, imaging guidance combined with large-format histopathology offers the most accessible technique for the routine clinical laboratory. The prevalence of ink migration into the interior of resection specimens and its influence on margin reporting is unknown; it may contribute to the underreporting of margin width in some cases. A fair question is whether a more thorough approach to margin evaluation resulting from LBP results in a higher incidence of close or inadequate surgical margins. In this paper, the opposite was found to be the case (Table 5). As an isolated undertaking, an LBP program would hypothetically result in the reporting of close or positive margins with greater frequency. In contrast, this paper describes an improvement in margin status and reexcision rate. The reported outcomes occurred, not from a large-format program in isolation, but from an LBP program fully integrated into an interdisciplinary breast center. Meticulous interdisciplinary presurgical planning with post-surgical outcome analysis and correlation of pre- and postsurgical imaging studies routinely occurred in a context of large-format pathology mapping of tumor extent and margin proximity. Optimized in this way, large-format-imaging correlations can influence not only the selection of surgical procedure, but the extent and volume of breast tissue removed to follow the anatomic distribution of cancer in the breast. Imagers become more conversant with the biologic behavior of breast cancer subtypes and their varied appearance in imaging studies, particularly MRI. Surgeons likewise gain confidence in the interpretation and implications of presurgical imaging studies. It seems likely that including a breast surgeon in the enhanced imaging large-format correlation process can improve the cross-disciplinary understanding of case-specific nuances in cancer distribution and optimize utilization of presurgical imaging studies to plan breast-conserving surgical procedures. 

The present-day clinical reliance on nonstandardized measurements of DCIS extent and proximity of DCIS to surgical margins to guide management decision making should raise theoretical and practical concerns. Most breast pathologists are aware of the challenges faced in the gross specimen identification of DCIS, the difficulty in measuring overall DCIS extent in resections and the practical necessity of limiting the number of sections taken from surgical specimens. As a result, the resulting data presented in the literature is a confusing blend of methodologies and is neither comparable across studies nor is it applicable to individual practice environments. The implication for future investigative work is that nonstandardized pathologist-generated breast cancer data will continue to obfuscate clinical research. All of these limitations existed well before the advent of breast conservation surgery, but their relevance is greater today. Many clinical decisions, such as whether to offer breast conservation, surgical reexcision, or radiation therapy, are based on pathologic parameters not deemed relevant to report a generation ago. In the present era of screen-detected breast cancer in asymptomatic women, the unnecessary limitations posed by traditional examination methods are a relevant concern not only for the occult component of a symptomatic or palpable lesion (as they undoubtedly were in the past), but also for the entire neoplastic process involving some patients' breasts.

Pathologists are understandably reluctant to intentionally increase costs and effort associated with specimen processing and reporting, especially in an environment of fixed reimbursement. Objectively, the incremental direct costs associated with the LBP program (Table 9) are moderated by a reduction in low-yield, random histopathology sections. Expressed as dollar expense per unit surface area examined, the technical costs of conventional and large section pathology are quite similar (Table 8). With a targeted, imaging-assisted specimen examination protocol, the pathologic reporting of clinically relevant data becomes more focused and efficient. The additional expense associated with an LBP program must be viewed in the broader context of interdisciplinary improvements in clinical efficiency and compassionate care and not as the sole burden of the pathology laboratory or department. The clinical consequences of improved margin status and lower reexcision rates for individual patients are readily apparent; viewed from the perspective of enhanced cost-efficiency on a national or global scale the gains could be substantial. 

## 5. Conclusions

This paper describes the successful incorporation of a large-format breast pathology program into an existing comprehensive breast care center based on a non-profit health system in the United States. From its inception, the program was designed to enhance the bidirectional information flow among breast imagers, surgeons, and pathologists. A goal of the initiative was to provide the breast team members with a more precise characterization of breast cancer attributes relevant to prognosis and clinical management through improved imaging, pathology, and clinical correlation. These measures were deemed appropriate in view of the increasing clinical reliance upon pathology-reported breast cancer attributes, wherein relatively small increments in reported tumor variables can translate into significant changes in clinical management and perceived prognosis. The experience derived from the development of this program indicates it is feasible and desirable for community hospital-based pathology and breast imaging departments to adopt these processes for the benefit of all patients in a comprehensive, interdisciplinary breast center.

## Figures and Tables

**Table 1:** Surgical margins: persistent management questions.

(i) Why does breast cancer recur after breast conservation surgery (BCS) with negative margins?	
(a) Corollary: why is carcinoma present in re-excisions/mastectomies performed following excisions with negative margins?	
(ii) What margin width is necessary to consider surgical management complete for DCIS and invasive carcinoma?	
(iii) Why are re-excisions of breast performed following BCS with positive margins often negative for residual cancer?	
(iv) Why does breast cancer locally recur following mastectomy when the mastectomy margins are negative?	
(v) Can extent or grade of DCIS predict recurrence when BCS margins are negative?	

**Table 2:** Summary of limitations of conventional pathologic technique in the diagnosis and reporting of breast carcinomas in breast-conserving surgical specimens.

(i) Gross inspection and palpation have insufficient sensitivity to guide section submission of imaging-only detected neoplasia, including DCIS and multifocal invasive carcinoma	
(ii) Complete imaging data, including MRI features are not available to pathologists at time of specimen evaluation	
(iii) Spatial 3D integrity of the specimen is lost through sectioning, section submission and histopathologic examination	
(iv) Margin evaluation is often reliant on gross inspection and palpation, even for imaging-only detected disease	
(v) Lack of standardization of methods to measure DCIS extent, multifocality, and margins	
(vi) Suboptimal correlation between pathology and pre-surgical imaging studies	

**Table 3:** Required program elements of the image-assisted large-format breast pathology initiative.

(i) Planning-phase knowledge base and budget development	
(ii) Specialized large-format/MRI interdisciplinary conference	
(iii) Surgical pathology gross specimen protocol	
(iv) Breast imaging department specimen radiography protocol	
(v) Histopathology laboratory large-format procedures	
(vi) Pathologist signout and reporting procedures	
(vii) Comprehensive breast cancer database development	
(viii) Training of pathologists, histopathology staff, breast imagers, and radiologic technologists	

**Table 4:** Percent of surface area available for conventional histopathologic evaluation of surgical margin status. Hypothetical 8.0 cm diameter lumpectomy specimen with one 2.0 cm × 2.0 cm tissue block removed for each of six axial margins. Specimen surface area = 201 cm^2^.

Examination method	Total surface margin area examined (cm^2^)	Percent of specimen surface area
En-face, six 2 × 2 cm sections	24 cm^2^	12%
Perpendicular, six sections 2 cm of arc × 0.3 cm thick*	3.6 cm^2^	1.8%
Perpendicular, six sections 2 cm of arc × 4 microns thick**	0.048 cm^2^	0.02%

*Assumes 6 tissue blocks 2 cm × 2 cm measuring 3 mm thickness each, maximum amount of tissue available for examination in the paraffin block.

**Assumes 6 tissue blocks 2 cm × 2 cm with final section thickness on microscopic slide of 4 microns.

**Table 5:** Comparison of 135 consecutive breast conserving surgical specimens with DCIS. Conventional pathology without MRI (CP) and large format breast pathology with MRI (LBP).

	CP *n* = 43	LBP *n* = 92
One or more of six margins measuring 0–4 mm	20 (47%)	22 (24%)
One or more of six margins measuring 0–9 mm	23 (54%)	31 (34%)
Re-excised after breast conservation	14 (32%)	11 (12%)
Volume of breast conservation specimen, median (cm^3^)	97.1	191.2
Breast conservation rate	65%	63%

**Table 6:** Extent measurements of duct carcinoma *in situ*. Comparison of 462 consecutive breast conserving surgical specimens. Conventional pathology without MRI (CP) versus large format breast pathology with MRI (LBP).

DCIS Extent, mm median (range)	CP *n* = 250	LBP *n* = 212
Histopathology	6.1 (1–24)	29.1 (1–125)
Mammography	11.0 (0–69)	12.0 (1–68)
MRI	Not performed	27.0 (2–113)

**Table 7:** Average number of conventional tissue blocks per case, year 2004-2005. Comparison between conventional pathology (CP) and LBP Program (LBP). *N* = 100.

CP	LBP
Lumpectomy, mean (range) 14.9 (7–33) *n* = 28	4.9 (3–12) *n* = 22
Mastectomy, mean (range) 13.0 (6–31) *n* = 28	6.9 (5–11) *n* = 22

**Table 8:** Direct labor and material expense per finished slide excluding amortization, year 2005. Comparison of conventional and large-format slides. $USD. *N* = 50 cases.

Slide type	Labor and materials per slide ($USD)	Approximate maximum surface area (cm^2^)	($USD)/cm^2^
Conventional	3.22	8.0	0.40
Large Format	38.22	80.0	0.48

**Table 9:** Imaging-guided large-format breast pathology net incremental technical expense per case. $USD, 2005.

Lumpectomy/excision (*n* = 20)	$USD
Increased expense from large-format section	38.22
Savings from reduction in conventional slides/case (mean reduction = 10 slides)	<32.20>
Net expense increase per case	6.02

Mastectomy (*n* = 20)	

Increased expense from large-format section	38.22
Savings from reduction in conventional slides/case (mean reduction = 6.1 slides)	<19.64>
Net expense increase per case	18.58

## References

[1] Duffy SW, Tabár L, Chen HH (2002). The impact of organized mammography service screening on breast carcinoma mortality in seven Swedish Counties: a collaborative evaluation. *Cancer*.

[2] Duffy SW (2006). Reduction in breast cancer mortality from organized service screening with mammography—1. Further confirmation with extended data. *Cancer Epidemiology Biomarkers and Prevention*.

[3] Tabar L, Yen MF, Vitak B, Tony Chen HH, Smith RA, Duffy SW (2003). Mammography service screening and mortality in breast cancer patients: 20-year follow-up before and after introduction of screening. *Lancet*.

[4] Coburn NG, Chung MA, Fulton J, Cady B (2004). Decreased breast cancer tumor size, stage, and mortality in Rhode Island: an example of a well-screened population. *Cancer Control*.

[5] Tabár L, Smith RA, Vitak B (2003). Mammographic screening: a key factor in the control of breast cancer. *Cancer Journal*.

[6] Morrow M, Schmidt RA, Bucci C (1998). Breast conservation for mammographically occult carcinoma. *Annals of Surgery*.

[7] Fitzal F, Gnant M (2006). Breast conservation: evolution of surgical strategies. *Breast Journal*.

[8] Fisher B, Bauer M, Margolese R (1985). Five-year results of a randomized clinical trial comparing total mastectomy and segmental mastectomy with or without radiation in the treatment of breast cancer. *New England Journal of Medicine*.

[9] Fisher B, Anderson S, Bryant J (2002). Twenty-year follow-up of a randomized trial comparing total mastectomy, lumpectomy, and lumpectomy plus irradiation for the treatment of invasive breast cancer. *New England Journal of Medicine*.

[10] Bijker N, Meijnen P, Peterse JL (2006). Breast-conserving treatment with or without radiotherapy in ductal carcinoma-in-situ: ten-year results of european organisation for research and treatment of cancer randomized phase III trial 10853—a study by the EORTC breast cancer cooperative group and EORTC radiotherapy group. *Journal of Clinical Oncology*.

[11] Swanson GP, Rynearson K, Symmonds R (2002). Significance of margins of excision on breast cancer recurrence. *American Journal of Clinical Oncology*.

[12] Scopa CD, Aroukatos P, Tsamandas AC, Aletra C (2006). Evaluation of margin status in lumpectomy specimens and residual breast carcinoma. *Breast Journal*.

[13] Dillon MF, Hill ADK, Quinn CM, McDermott EW, O’Higgins N (2006). A pathologic assessment of adequate margin status in breast-conserving therapy. *Annals of Surgical Oncology*.

[14] DiBiase S, Komarnicky L, Schwartz G (1998). The number of positive margins influences the outcome of women treated with breast preservation for early stage breast carcinoma. *Cancer*.

[15] Schnitt S, Abner A, Gelman R (1994). The relationship between microscopic margins of resection and the risk of local recurrence in patients with breast cancer treated with breast-conserving surgery and radiation therapy. *Cancer*.

[16] Holland R, Connolly JL, Gelman R (1990). The presence of an extensive intraductal component following a limited excision correlates with prominent residual disease in the remainder of the breast. *Journal of Clinical Oncology*.

[17] Anscher MS, Jones P, Prosnitz LR (1993). Local failure and margin status in early-stage breast carcinoma treated with conservation surgery and radiation therapy. *Annals of Surgery*.

[18] Spivack B, Khanna MM, Tafra L (1994). Margin status and local recurrence after breast-conserving surgery. *Archives of Surgery*.

[19] Kunos C, Latson L, Overmoyer B (2006). Breast conservation surgery achieving ≥2 mm tumor-free margins results in decreased local-regional recurrence rates. *Breast Journal*.

[20] Cheng L, Al-Kaisi NK, Gordon NH, Liu AY, Gebrail F, Shenk RR (1997). Relationship between the size and margin status of ductal carcinoma *in situ* of the breast and residual disease. *Journal of the National Cancer Institute*.

[21] Macdonald HR, Silverstein MJ, Lee LA (2006). Margin width as the sole determinant of local recurrence after breast conservation in patients with ductal carcinoma *in situ* of the breast. *American Journal of Surgery*.

[22] Silverstein M, Gierson E, Colburn W (1994). Can intraductal breast carcinoma be excised completely by local excision?. *Cancer*.

[23] MacDonald HR, Silverstein MJ, Mabry H (2005). Local control in ductal carcinoma *in situ* treated by excision alone: incremental benefit of larger margins. *American Journal of Surgery*.

[24] Holland PA, Gandhi A, Knox WF, Wilson M, Baildam AD, Bundred NJ (1998). The importance of complete excision in the prevention of local recurrence of ductal carcinoma *in situ*. *British Journal of Cancer*.

[25] Chan K, Knox W, Sinha G (2001). Extent of excision margin width required in breast conserving
surgery for ductal carcinoma *in situ*. *Cancer*.

[26] Douglas-Jones AG, Logan J, Morgan JM, Johnson R, Williams R (2002). Effect of margins of excision on recurrence after local excision of ductal carcinoma *in situ* of the breast. *Journal of Clinical Pathology*.

[27] Neuschatz AC, DiPetrillo T, Steinhoff M (2002). The value of breast lumpectomy margin assessment as a predictor of residual tumor burden in ductal carcinoma *in situ* of the breast. *Cancer*.

[28] Mullenix PS, Cuadrado DG, Steele SR (2004). Secondary operations are frequently required to complete the surgical phase of therapy in the era of breast conservation and sentinel lymph node biopsy. *American Journal of Surgery*.

[29] Rodriguez N, Diaz LK, Wiley EL (2005). Predictors of residual disease in repeat excisions for lumpectomies with margins less than 0.1 cm. *Clinical Breast Cancer*.

[30] MacAusland SG, Hepel JT, Chong FK (2007). An attempt to independently verify the utility of the Van Nuys Prognostic Index for ductal carcinoma *in situ*. *Cancer*.

[31] Tucker FL (2008). New era pathologic techniques in the diagnosis and reporting of
breast cancers. *Seminars in Breast Disease*.

[32] Campbell ID, Theaker JM, Royle GT (1991). Impact of an extensive *in situ* component on the presence of residual disease in screen detected breast cancer. *Journal of the Royal Society of Medicine*.

[33] Tot T, Tabár L, Dean PB (2000). The pressing need for better histologic-mammographic correlation of the many variations in normal breast anatomy. *Virchows Archiv*.

[34] Lester S, Bose S, Chen Y (2009). Protocol for the examination of specimens from patients with
ductal carcinoma *in situ* of the breast. *Archives of Pathology and Laboratory Medicine*.

[35] Grin A, Horne G, Ennis M, O’Malley FP (2009). Measuring extent of ductal carcinoma *in situ* in breast excision specimens. *Archives of Pathology and Laboratory Medicine*.

[36] Dadmanesh F, Fan X, Dastane A, Amin MB, Bose S (2009). Comparative analysis of size estimation by mapping and counting number of blocks with ductal carcinoma *in situ* in breast excision specimens. *Archives of Pathology and Laboratory Medicine*.

[37] Dillon MF, Mc Dermott EW, O’Doherty A, Quinn CM, Hill AD, O’Higgins N (2007). Factors affecting successful breast conservation for ductal carcinoma *in situ*. *Annals of Surgical Oncology*.

[38] Menell JH, Morris EA, Dershaw DD, Abramson AF, Brogi E, Liberman L (2005). Determination of the presence and extent of pure ductal carcinoma *in situ* by mammography and magnetic resonance imaging. *Breast Journal*.

[39] Holland R, Veling SHJ, Mravunac M, Hendriks JHCL (1985). Histologic multifocality of Tis, T1-2 breast carcinomas: implications for clinical trials of breast-conserving surgery. *Cancer*.

[40] Kato T, Kimura T, Ishii N (2000). Pathologic evaluation of surgical margins and local recurrences after breast-conserving surgery without irradiation. *World Journal of Surgery*.

[41] Sigal-Zafrani B, Lewis JS, Clough KB (2004). Histological margin assessment for breast ductal carcinoma *in situ*: precision and implications. *Modern Pathology*.

[42] Mai KT, Perkins DG, Mirsky D (2003). Location and extent of positive resection margins and ductal carcinoma *in situ* in lumpectomy specimens of ductal breast carcinoma examined with a microscopic three-dimensional view. *Breast Journal*.

[43] Ichihara S, Suzuki H, Kasami M (2001). A new method of margin evaluation in breast conservation surgery using an adjustable mould during fixation. *Histopathology*.

[44] Berg WA, Gutierrez L, NessAiver MS (2004). Diagnostic accuracy of mammography, clinical examination, US, and MR imaging in preoperative assessment of breast cancer. *Radiology*.

[45] Schouten van der Velden AP, Boetes C, Bult P, Wobbes T (2006). The value of magnetic resonance imaging in diagnosis and size assessment of *in situ* and small invasive breast carcinoma. *American Journal of Surgery*.

[46] Chung A, Saouaf R, Scharre K, Phillips E (2005). The impact of MRI on the treatment of DCIS. *American Surgeon*.

[47] Houssami N, Ciatto S, Macaskill P (2008). Accuracy and surgical impact of magnetic resonance imaging in breast cancer staging: systematic review and meta-analysis in detection of multifocal and multicentric cancer. *Journal of Clinical Oncology*.

[48] Solin LJ (2010). Counterview: pre-operative breast MRI (magnetic resonance imaging) is not recommended for all patients with newly diagnosed breast cancer. *Breast*.

[49] Tot T, Tabar L, Dean P (2002). *Practical Breast Pathology*.

[50] Tot T (2007). Clinical relevance of the distribution of the lesions in 500 consecutive breast cancer cases documented in large-format histologic sections. *Cancer*.

[51] Wellings SR, Jensen HM, Marcum RG (1975). An atlas of subgross pathology of the human breast with special reference to possible precancerous lesions. *Journal of the National Cancer Institute*.

[52] Jackson PA, Merchant W, McCormick CJ, Cook MG (1994). A comparison of large block macrosectioning and conventional techniques in breast pathology. *Virchows Archiv*.

[53] Foschini MP, Flamminio F, Miglio R (2007). The impact of large sections on the study of *in situ* and invasive duct carcinoma of the breast. *Human Pathology*.

[54] Foschini MP, Righi A, Cucchi MC (2006). The impact of large sections and 3D technique on the study of lobular *in situ* and invasive carcinoma of the breast. *Virchows Archiv*.

[55] Biesemier KW, Alexander MC (2006). Enhancement of mammographic-pathologic correlation utilizing large
format histology for malignant breast disease. *Seminars in Breast Disease*.

[56] Méchine-Neuville A, Chenard MP, Gairard B, Mathelin C, Bellocq JP (2000). Large section technique in breast pathology an appropriate response to breast-conserving surgery. *Annales de Pathologie*.

[57] Silverstein M, Recht A, Harms S (2005). Image-detected breast cancer: state of the art diagnosis and
treatment. *Journal of the American College of Surgeons*.

[58] Idowu M, Hardy L, Souers R (2012). Pathologic diagnostic correlation with breast imaging findings. A
college of american pathologists Q-probes study of 48 institutions. *Archives of Pathology and Laboratory Medicine*.

